# Primary surgery of subcondylar mandibular fracture using patient-specific implant: the Helsinki protocol

**DOI:** 10.1007/s10006-025-01338-2

**Published:** 2025-01-24

**Authors:** Mikko Saloniemi, Malla Salli, Valtteri Lehtinen, Johanna Snäll

**Affiliations:** 1 Department of Oral and Maxillofacial Diseases, Helsinki University and Helsinki University Hospital, Helsinki, Finland; 2 Oral and Maxillofacial Surgery Ward S7A, Meilahti Bridge Hospital, Helsinki, Finland

**Keywords:** Patient-specific modeling, Prostheses and implants, Mandibular fractures, Fracture osteosyntheses

## Abstract

**Purpose:**

Preoperative virtual planning and osteosynthesis with patient-specific implants (PSIs) have become a quotidian approach to many maxillofacial elective surgery setups. When a process is well-organized, a similar approach can be harnessed to serve the needs of exact primary reconstructions, especially in midfacial trauma cases. PSI osteosynthesis of the mandible is, however, more challenging because a mirror technique of the facial sides is often unreliable due to inherent lack of symmetry, and movement of the mandible increases the risk of loosening of the osteosynthesis. The purpose of this study was to present clinical results of the Helsinki protocol concept of utilizing PSIs in the primary surgery of unilateral mandibular subcondylar fractures as the first publication on the subject.

**Methods:**

A single-center study of a new Helsinki protocol is presented for surgical treatment of subcondylar mandibular fractures using patient-specific, titanium-milled repositator plates. Ten patients with dislocated subcondylar mandibular fractures received surgery and osteosynthesis with PSI via a retromandibular approach.

**Results:**

Clinical and radiological outcomes were excellent; none of the patients had fixation-related major complications or developed postoperative malocclusion.

**Conclusions:**

Study results show that the Helsinki protocol, treating mandibular condylar fractures primarily with PSI plates, is a viable treatment option.

## Introduction

Mandibular condylar process fractures are one of the most common types of mandibular fractures, accounting for 28–45% of the cases [1–3]. They can be subclassified based on their anatomical level as condylar head, neck, or subcondylar fractures [4]. Displaced mandibular condylar fractures are commonly treated with open reduction and internal fixation (ORIF) to re-establish occlusal contacts and posterior facial height, restore the function of the temporomandibular joints, and reduce pain, as ORIF has shown superior postoperative functional outcomes to closed treatment [5, 6]. However, from a technical perspective, gaining adequate visibility to the surgical field may be difficult in complex and severely dislocated condyle fractures without using wide surgical approaches. This, again, may increase the risk of complications, especially facial nerve dysfunctions [7–9]. Therefore, a need exists for technical innovations that would aid complex fracture reduction and fixation, allowing use of minimal surgical incisions and intraoperative manipulation and providing stable fracture fixation.

Computed tomography (CT) is today´s standard of care in facial trauma diagnostics. In addition to its accurate ability to detect traumatic findings, CT enables virtual surgical planning and design of patient-specific implants (PSIs). PSIs elevate the anatomical fit of osteosynthesis material to an outstanding level compared with stock plates [10] and allow concurrent use of plating material as a surgical repositioning guide [11, 12]. In maxillofacial surgery today, PSIs are commonly used in elective setups, such as orthognathic surgery and some primary trauma surgery, mainly orbital fractures, with excellent results [10, 11].

The main goal of this study was to present clinical results of PSIs in the primary surgery of unilateral mandibular subcondylar fractures as the first publication on the subject. A single-plate system was used to aid the visualization of the anatomical fracture reduction and to allow sufficient primary stability and exact implant placement. The hypothesis was that PSIs are a technically helpful treatment method also in displaced subcondylar mandibular fractures, providing superior postoperative outcomes to stock plates.

## Materials and methods

### Study design and inclusion criteria

A retrospective study of all patients operated on for unilateral condylar fractures at the Department of Oral and Maxillofacial Diseases, Helsinki University Hospital, Finland, between January 2018 and June 2023 was conducted. Data of patients with a PSI used for primary reconstruction of a displaced unilateral subcondylar mandibular fracture with available preoperative CT imaging data were included. Data were retrospectively collected from electronic patient records.

### Study variables

Pre- and perioperative variables included age, sex, delay from injury to surgery, type of condylar fracture, occurrence of other associated injuries, injury mechanism, surgical approach, and duration of follow-up. Postoperative outcomes were assessed based on clinical and radiological evaluation during the follow-up period. Clinical outcomes included occlusion, jaw function, prolonged pain, facial nerve dysfunction, wound dehiscence, salivary fistulas, infections, and need for revision surgery. Clinical postoperative complications were graded using the Clavien-Dindo classification for surgical complications [13].

### Postoperative follow-up and radiological imaging

Postoperative imaging included dental panoramic tomography imaging and an angled anteroposterior radiograph of the skull to visualize the mandibular condyles in anteroposterior projection (Towne view) or CT or cone-beam computed tomography (CBCT) scans during hospital stay. Patients were followed up after one week, 5 weeks, and 3 months, and later if necessary. Postoperative imaging was performed immediately after surgery and at 3 months postoperatively. When CT or CBCT was available, a comparative assessment between preoperative virtual position of the fracture and the plate and the postoperative position was conducted.

### Virtual planning and manufacturing of PSIs

**Fig. 1:**
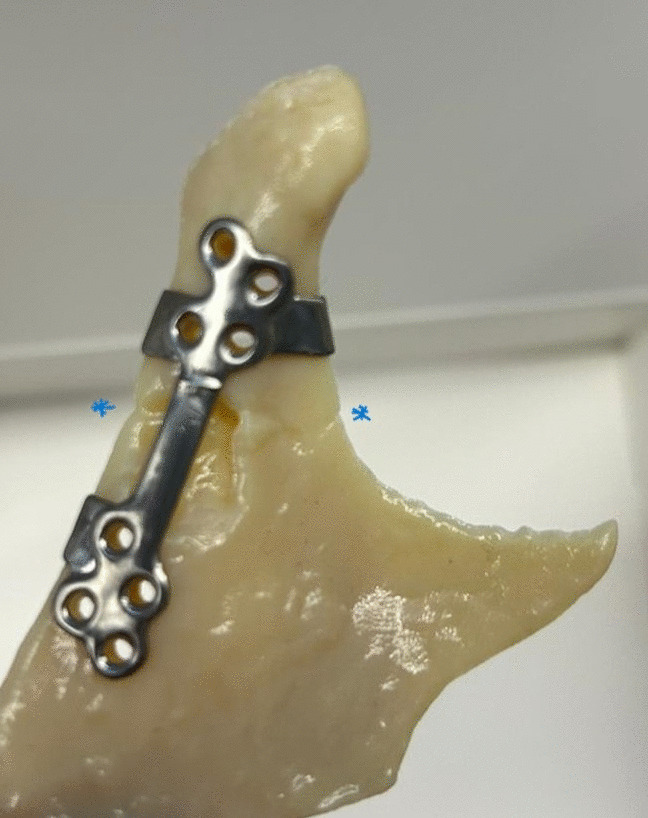
Patient-specific implant on a model, with the fracture line after virtual reposition depicted in the region between two asterisks (*). Fixation screw vectors were designed in a non-parallel way to create maximal support through the masticatory movement range. Small indentations were designed to indicate the fracture edges. Small tabs and hooks were placed to indicate anatomical landmarks, such as the coronoid notch and condylar neck, to aid in placement of fixation plates and anatomical reduction of fractures. Screw holes (2.0 mm) for non-locking screws (KLS Martin^®^, Depuy Synthes^®^, Stryker^®^) were designed for at least three screws on both sides of the fracture line and to allow non-parallel fixation vectors

**Fig. 2:**
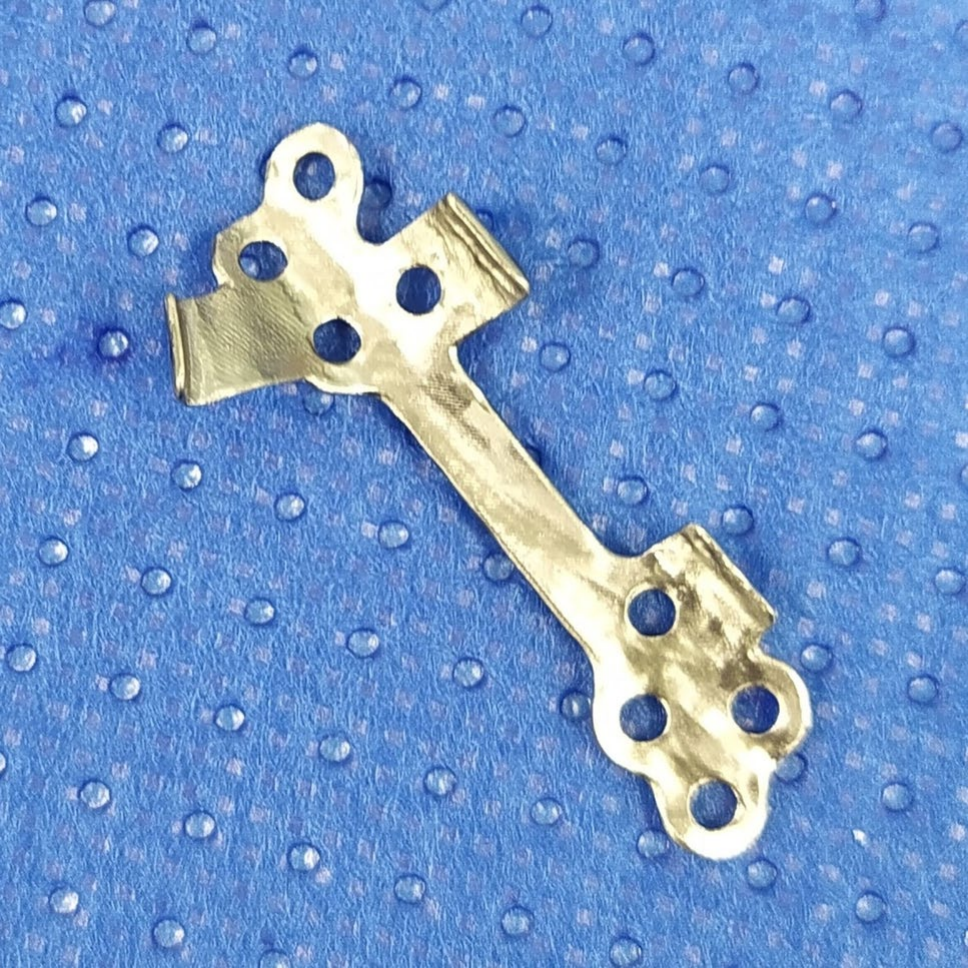
Patient-specific implant with the bone-facing surface upwards. Note the small tabs and hooks ensuring accurate fit

Preoperative radiological assessments were conducted with a high-resolution 16-slice CT scanner (Siemens or GE Medical Systems), and images were exported in DICOM (Digital Imaging and Communications in Medicine) format. Virtual planning of the surgery and plates was conducted by one of the authors (JS) and by a biomedical engineer using the PlanmecaProModel™ system (Planmeca Ltd., Helsinki, Finland). Proximal and distal fracture fragments were segmented from CT images into surface meshes, and fracture reduction was performed virtually. Virtual fracture reduction was based on the surface topography and visual continuity of the fracture surfaces rather than on virtual occlusion or mirroring protocols. The surgeon confirmed the final reduced fracture position on which the PSI was designed. PSIs were Computer Numerical Control (CNC) -milled from grade II titanium alloy blocks with non-locking screw apertures by Planmeca Ltd. The implants were preoperatively heat-sterilized. A sample of PSI design is provided in Figs. 1 and 2.

### Surgery

**Fig. 3:**
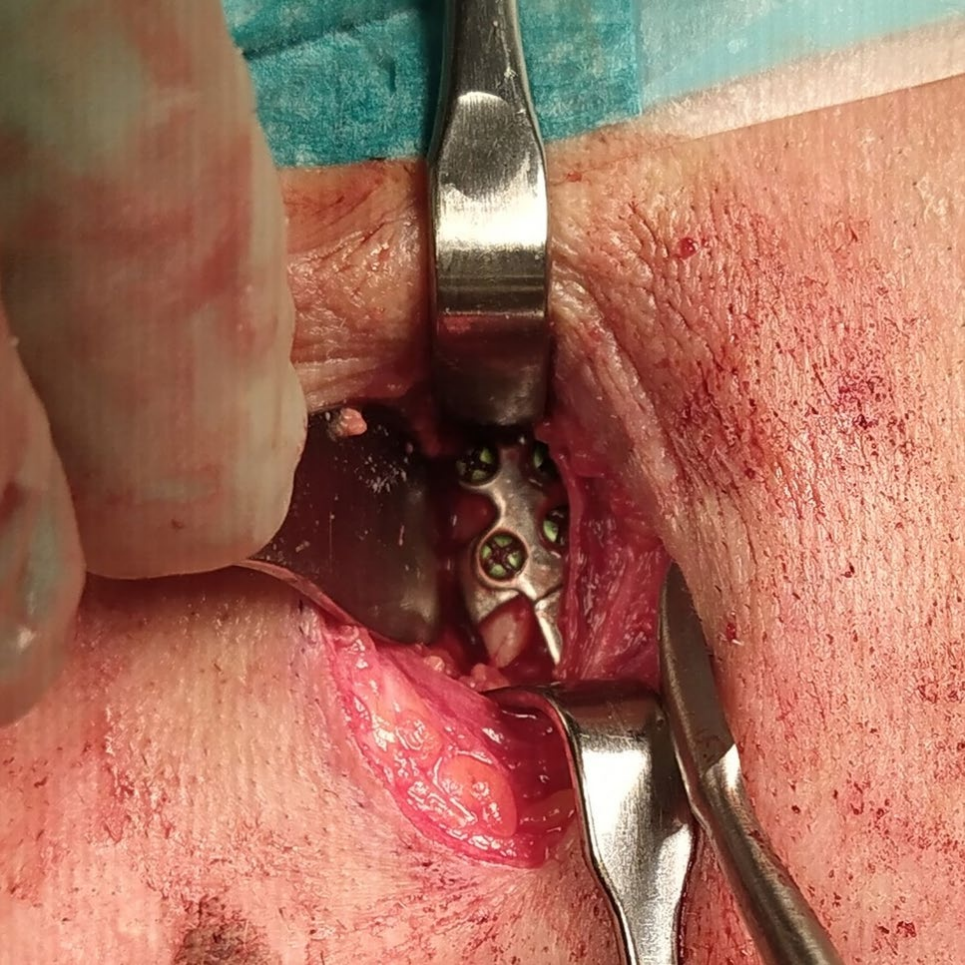
Retromandibular access and intraoperative view. Note the slight line on the PSI indicating the fracture level

Surgeries were performed under general anesthesia via a retromandibular approach as illustrated in Fig. 3. No occlusal splinting or intermaxillary fixation was used during occlusion-based reposition. Two maxillofacial surgeons participated in all surgeries. The final screw fixations were performed while the occlusion was kept in manual reduction. One of the surgeons was an experienced senior surgeon in all cases. All patients received antibiotic prophylaxis. No intraoperative radiological imaging or navigation was used.

### Statistical analyses

Data were tabulated and analyzed using MS Excel (Microsoft Inc.). Due to the small number of cases, statistical analyses were not performed, and results are shown as means and ranges of values.

## Results

**Fig. 4:**
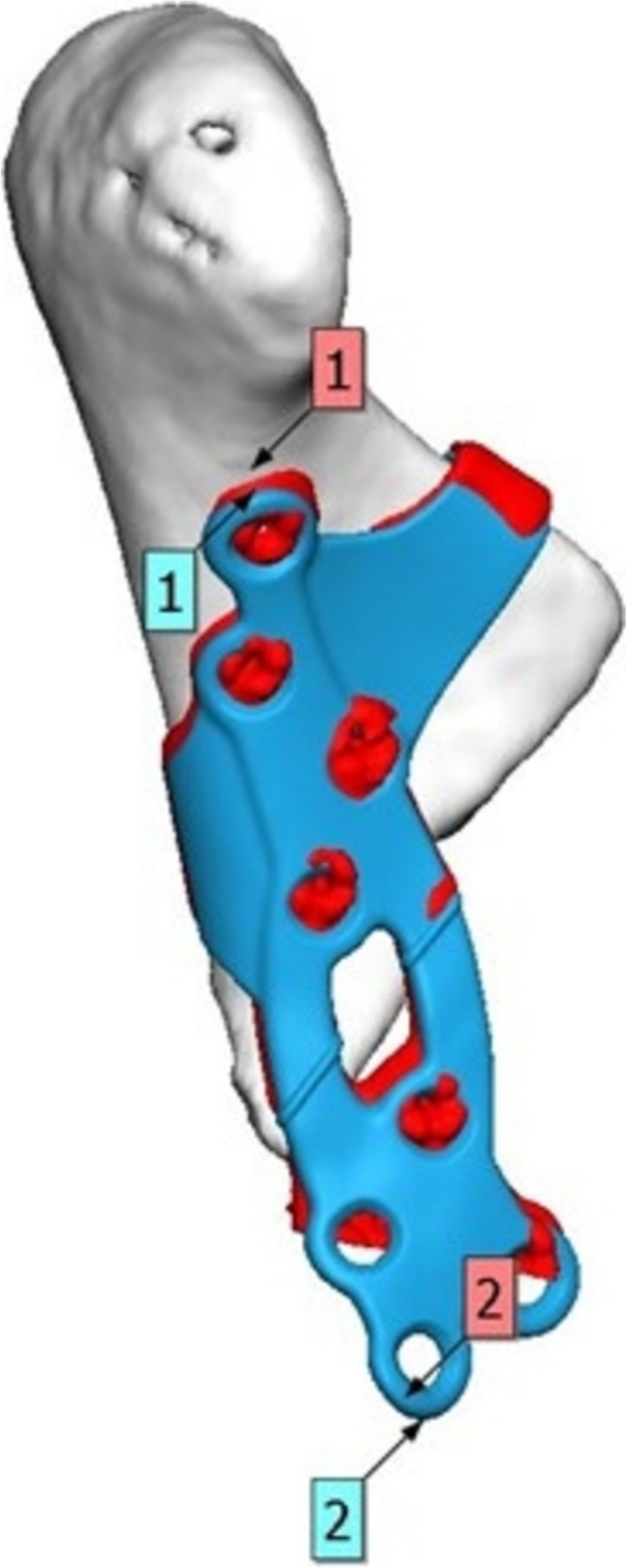
Postoperative computed tomography (CT) or cone beam computed tomography (CBCT) data available for three patients. 3D postoperative analysis was performed for these patients. It revealed excellent fit of the patient-specific implant on the condyles, with a mean discrepancy of 0.2 mm horizontally, 0.1 mm anteroposteriorly, and 0.6 mm vertically (1) at the most cranial screw hole. The caudal aspect (2) of the plate differed from the planned position by 1.2 mm horizontally, 1.8 mm anteroposteriorly, and 0.8 mm vertically

Ten patients with displaced subcondylar mandibular fractures were treated with ORIF with PSIs during the study period. Sample characteristics are summarized in Table 1. All fractures were classified as low condylar neck fractures. Surgical indications were changed occlusion with or without open bite and/or shortening of ramus height. Four patients had an additional facial bone fracture associated with the condylar fracture. The virtual planning process, including plate delivery, occurred within 24 h of admittance to hospital. Mean delay between injury and surgery was 11.6 days, and mean follow-up time was 210.2 days. PSIs were fixated with 4–8 (mostly 8) screws. Postoperative implant positions were accurate (Fig. 4.)

**Table 1:** Background and clinical variables of mandibular fracture patients

Variable	Value
Sample size	10
Age	Mean age 52.85 (SD 15.52) years
Sex	7 (70%) male, 3 (30%) female
Smoking	5 (50%) smoking, 5 (50%) non-smoking
Injury mechanism	Falling, *n* = 7
	Assault, *n* = 2
	Bicycle accident, *n* = 1
Indication(s) for surgery**	Open bite, *n* = 6
	Changed occlusion, *n* = 10
	Shortened ramus, *n* = 6
	Deviation in mouth opening, *n* = 2
Fracture type	Simple, *n* = 7
	Comminuted, *n* = 3
Associated facial fractures	Orbital fracture, *n* = 1
	Zygomatic fracture, *n* = 1
	Mandibular parasymphysis fracture, *n* = 1
	External auditory canal fracture, *n* = 1
Time between injury and surgery	Mean 11.60 (SD 14.63) days
Number of fixation screws	Mean 6.88 (SD 1.33) screws
Length of follow-up	Mean 210.20 (SD 316.76) days
Maximal mouth opening***	Mean 39.44 (SD 14.32) mm

** all indications listed in patient records were listed

*** as measured at the last follow-up

As presented in Table 2, no major postoperative complications or requirements for reoperation occurred. A minor postoperative complication in two patients was radiological suspicion of loosening of screw fixation with no clinical implications. Reversible temporomandibular disc displacement was found in two patients during follow-up; both were treated non-surgically. All patients had satisfactory postoperative occlusion with no requirement for postoperative surgical or prosthodontic procedures. Jaw movements were graded as normal in all patients.

**Table 2:** Surgical complications graded with Clavien-Dindo classification^13^ and minor complications not requiring surgical intervention

Clavien-Dindo classification	No. of complications in 10 patients
Grade I*	
Minor occlusal disturbance	2**
Wound dehiscence	0
Postoperative infection	0
Disc displacement	2
Suspected screw loosening	2***
Grade II	0
Grade III	0
Grade IV	0
Grade V	0

* Due to associated injuries sustained by some patients, Grade I complications were recorded only if clearly related to condylar fracture surgery

** 1 patient had subjective sensation of occlusal interference without any related objective findings, and 1 patient had imprecise yet balanced occlusion without subjective complaints

*** 2 patients had radiologically suspected minor screw loosening with no clinical significance

## Discussion

To our knowledge, this is the first publication describing the use of titanium-milled PSIs in the primary surgical treatment of unilateral, displaced subcondylar mandibular fractures. All surgeries were successful, as virtual reduction provided anatomically accurate fixation plates, and all patients had adequate postoperative occlusion with no major complications.

PSIs were planned based on regular trauma CT scans without a requirement for additional radiological examinations. Plate design took approximately 15 min of the surgeon´s time, and, due to local planning and manufacturing, the implants were manufactured and delivered within 24 h if needed. Therefore, PSI planning and manufacturing did not cause any delays in surgical treatment. The previous literature regarding surgical treatment of mandibular condylar fractures is not univocal in quality or conclusions. The most acknowledged fixation method comprises two plates, of which the posterior is aligned along the compressive strain and the anterior against the tensile strain along the mandibular notch [14, 15]. Other types of stock plates include single stock frame plates, referred to as “three-dimensional anatomical plates”, which may facilitate plate handling during surgery compared with the preeminent two-plate system. Moreover, theoretically, it may provide a more rigid fixation with reduced strain on the surrounding bone [16–18], but its clinical significance remains unclear [19]. Therefore, even after thorough finite element analysis (finite element method, FEM), no consensus regarding the best fixation method has been reached [20–22].

Postoperative complications of condylar fractures are quite rare. The most common complications include facial asymmetry, limitation or deviation during mouth opening or lateral excursion, malocclusion, facial nerve dysfunction, complications related to fixation hardware, surgical complications such as surgical site infection, salivary fistulas or sialocele formation, and hypertrophic scarring [6]. Facial nerve dysfunction is perhaps the most common complication, with a relative incidence of 0.3–28.6%, usually of a transient nature [6, 8, 9, 14, 23]. Surgical approaches entailing excessive manipulation of tissues or dissection of facial nerve branches are the most susceptible to facial nerve dysfunction [8, 9], which, as expected, is nonexistent in transoral approaches. Salivary fistulas are rare complications with a reported incidence of 1.11–2.3%. They are related to dissection of the parotid fascia during surgery and most often resolve during the first postoperative weeks [7, 14, 23]. Moreover, the rate of hardware complications, including breakage or torsion of plates and loosening of screws, lies between 1.8% and 12.2% [22–24]. These complications tend to occur in fracture fixations performed with a single miniplate, as opposed to other fixation methods [22, 24].

**Fig. 5:**
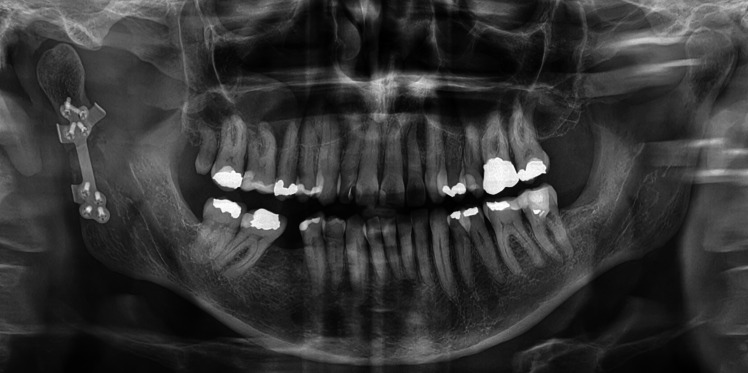
Postoperative dental panoramic tomography image after reduction and open fixation of the right condylar neck fracture with a patient-specific implant

Open reduction and internal fixation of condylar fractures are technically challenging due to the requirement to manipulate the fragments in a relatively small operative field with restricted visibility, which may easily lead to complications. Thus, there is a demand for a reliable and easy-to-install osteosynthesis device. The PSIs presented here were designed to aid the anatomical placement of the implant by adding the collar-style hook around the condylar neck as seen on Fig. 5., which also helps to manipulate the condylar fragment. In addition, the indentations and tabs of the implant were intended to increase the reliability of the fracture reduction by showing the relation of the planned plate position and the fracture line. Similar implant design has previously been successfully used also for condylar head fractures [12] and zygomaticomaxillary fractures [11]. Furthermore, divergent fixation vectors were designed to increase the resistance of multiple force vectors in variable angles and minimize the risk of screws interfering with each other. A diverging screw pattern has been shown to be superior to parallel or convergent screw vectors in various clinical situations ranging from malleolar fractures [25] to tibial osteotomies [26] to radial head fractures [27] with a transfer of strain to the surrounding bone [20]. The use of PSI did not require any changes to the standard retromandibular approach. The size of the implant corresponds to the size of the standard factory-made osteosynthesis plates used in the treatment of condylar fractures. The surgical approach may even be smaller when using PSI compared to the standard two-plate fixation strategy. No other incisions were used in our study, but PSI does not preclude the use of other approaches.

To conclude, this case series demonstrated the feasibility of single-plate PSIs to treat displaced, unilateral subcondylar fractures with generally excellent results. Postoperative implant positions were accurate and satisfactory occlusion was reconstructed in all patients without major complications. Weaknesses of this study are the retrospective design, which was based on electronic patient records, and the small size of the study group, restricting conclusive statistical analyses. Further FEM and biomechanical studies may offer more profound insight into the optimal design of PSIs.

## Conclusions

PSIs are a valid and reliable primary treatment option for displaced subcondylar fractures. They provide a precise anatomical fracture reduction and accurate postoperative occlusion. In addition, PSIs may solve the problems associated with hardware failure seen with other osteosynthesis methods and the technical difficulties related to the performance of the surgery. PSIs should be considered for wider use and further studies in primary surgery of subcondylar and other facial fractures.

## Data Availability

No datasets were generated or analysed during the current study.
